# Supplementary material for: Subnanometric Control of Coupling between WS2 Monolayers with a Molecular Spacer
Source: ACS Appl Mater Interfaces. 2025 Sep 11;17(38):53935–41. doi: 10.1021/acsami.5c12764 (PMC12464900; doi:10.1021/acsami.5c12764)
Supplement: Supplementary file 1 [file am5c12764_si_001.pdf]

Supporting Information

Subnanometric control of coupling between WS_2 monolayers with a molecular spacer

Sara A. Elrafei[1], Tom. T. C. Sistermans[2,3], Alberto G. Curto[1,2,3*]

[1] Department of Applied Physics and Eindhoven Hendrik Casimir Institute, Eindhoven University of Technology, 5600 MB Eindhoven, The Netherlands

[2] Photonics Research Group, Ghent University-imec, 9052 Ghent, Belgium

[3] Center for Nano- and Biophotonics, Ghent University, 9052 Ghent, Belgium

* Corresponding author: A.G.Curto@TUe.nl

Contents

Supporting Section S1. Doping of monolayer WS_2

Supporting Section S2. Permittivity and transmission spectrum of monolayer WS_2

Supporting Section S3. Atomic force microscopy

Supporting Section S4. Hyperspectral PL imaging

Supporting Section S1. Doping of monolayer WS_2

The adsorption of 7,7,8,8-tetracyanoquinodimethane (TCNQ) molecules on a monolayer WS_2 is particularly interesting due to the induced charge transfer. It provides *p*-type doping and brings the inherently *n*-type WS_2 crystal closer to an intrinsic semiconductor, enhancing its PL quantum efficiency without affecting the exciton energy and linewidth. The molecular spacer is prepared by spin-casting a TCNQ solution in methanol on a WS_2 monolayer on PDMS. Here, we compare the PL spectra of single WS_2 monolayers with and without TCNQ doping. A TCNQ concentration of 1 mM leads to a 2.5-fold enhancement in the PL intensity of the monolayer (Supporting Figure S1a). The enhancement of PL intensity in WS_2 monolayers exhibits a direct relationship with the concentration of TCNQ dopants; an increase in TCNQ concentration leads to a corresponding rise in PL intensity. This trend persists until saturation, at which point the PL intensity stabilizes despite further increases in TCNQ concentration (Supporting Figure S1b). The PL enhancement is due to the shift in recombination dynamics from negative trions to excitons.

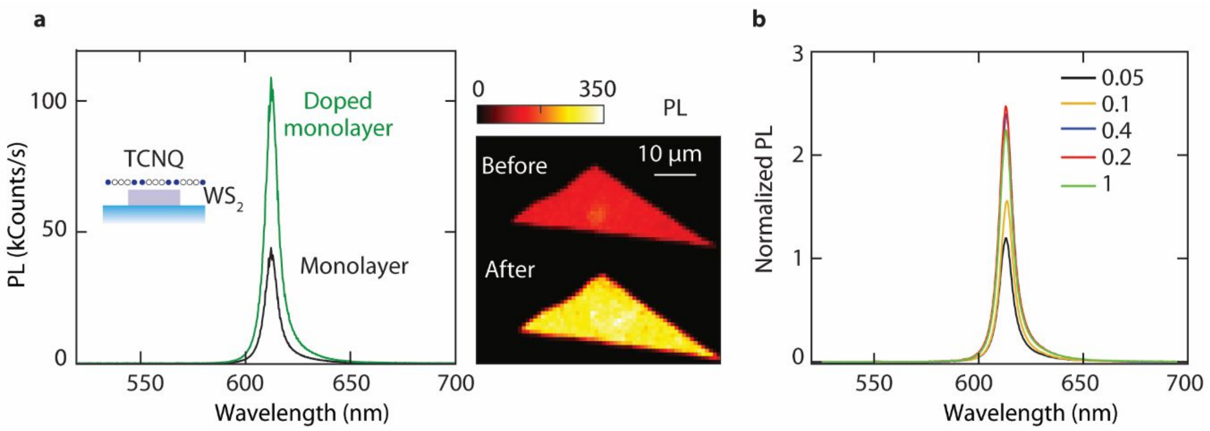

Supporting Figure S1 | Doping of monolayer WS_2. a, PL spectrum of monolayer WS_2 on PDMS with and without TCNQ doping. PL maps illustrating the emission enhancement of a monolayer before and after doping. Inset: schematic of a monolayer WS_2 incorporating TCNQ at a concentration of 1 mM. **b**, Normalized PL intensity with increasing molecular concentration (in mM) of single WS_2 monolayers, calculated as the ratio of the peak intensities after/before doping for each monolayer.

Supporting Section S2. Permittivity and transmission spectrum of WS_2

We retrieve the in-plane permittivity of monolayer WS_2 by fitting the transmission spectrum using the transfer-matrix method and a superposition of 4 Lorentzian oscillators, namely:

$$\varepsilon(E) = \varepsilon_{\text{background}} + \sum_{i=1}^{i=4} \mathrm{f}_i/(E_{i,\,\text{exciton}}^2 - E^2 - i\gamma_i E),$$

where ε_B is a background permittivity, f_i is the oscillator strength of the exciton with subindex i, $E_{i,\text{ exciton}}$ is the corresponding exciton peak energy, γ_i is the linewidth of the exciton absorption band, and $E = \hbar\omega$ is the photon energy.

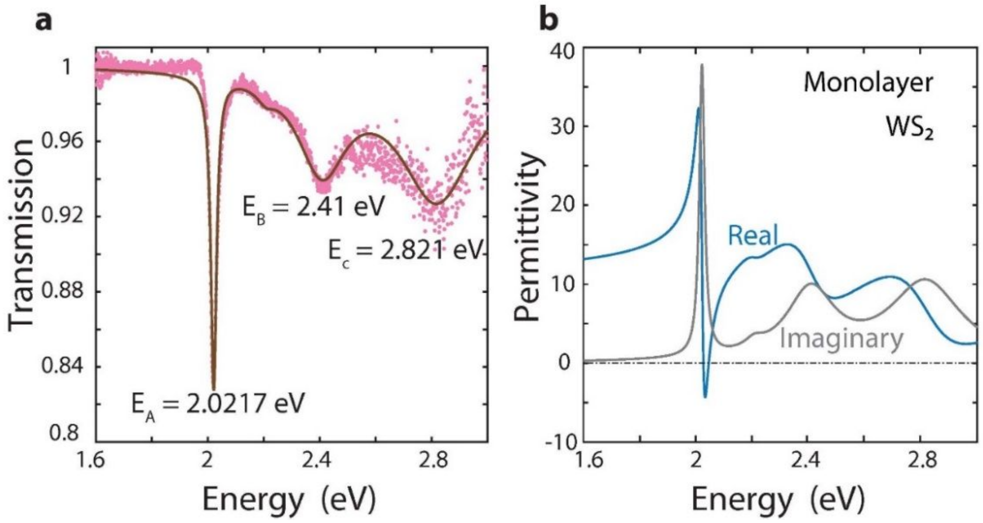

Supporting Figure S2 | Retrieval of the in-plane permittivity of monolayer WS_2. a, Experimental transmission spectrum of monolayer WS_2 on PDMS (pink) and fitted spectrum (brown). **b**, Retrieved in-plane permittivity of monolayer WS_2 obtained by fitting the transmission spectrum using the transfer-matrix method and a permittivity model with 4 Lorentzians.

Supporting Section S3. Atomic force microscopy

TCNQ molecules are suited to act as molecular spacers for monolayers due to their inherently planar structure, which ensures the deposition of a thin and uniform film and allow for efficient charge distribution and interaction. We verified the adsorption of TCNQ molecules onto the monolayers and confirm that there is no substantial inflicted damage to the monolayer due to spin coating using atomic force microscopy (Supporting Figure S3).

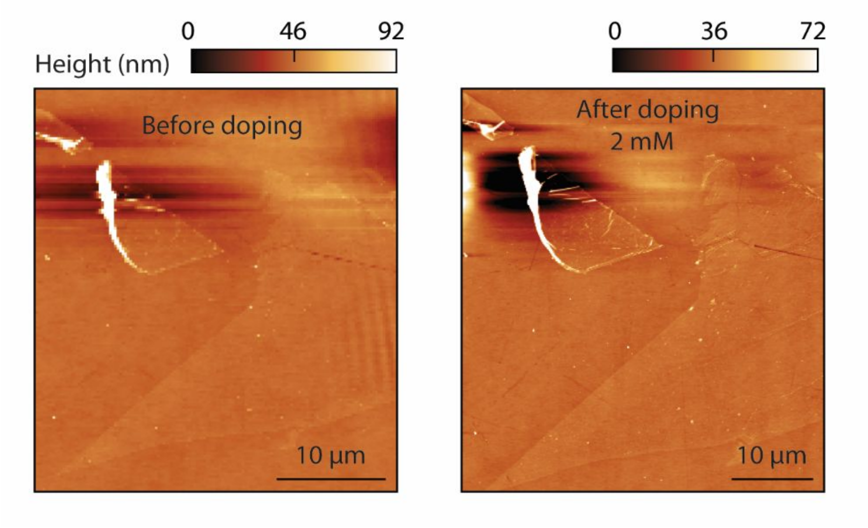

Supporting Figure S3 | AFM images of monolayer WS_2 on amorphous quartz. Comparison before and after TCNQ doping.

To calibrate the TCNQ spacer thickness, we carried out AFM imaging of TCNQ films spin cast on amorphous quartz. The samples were subsequently scratched to create a step corresponding to the molecular layer thickness (Supporting Figure S4).

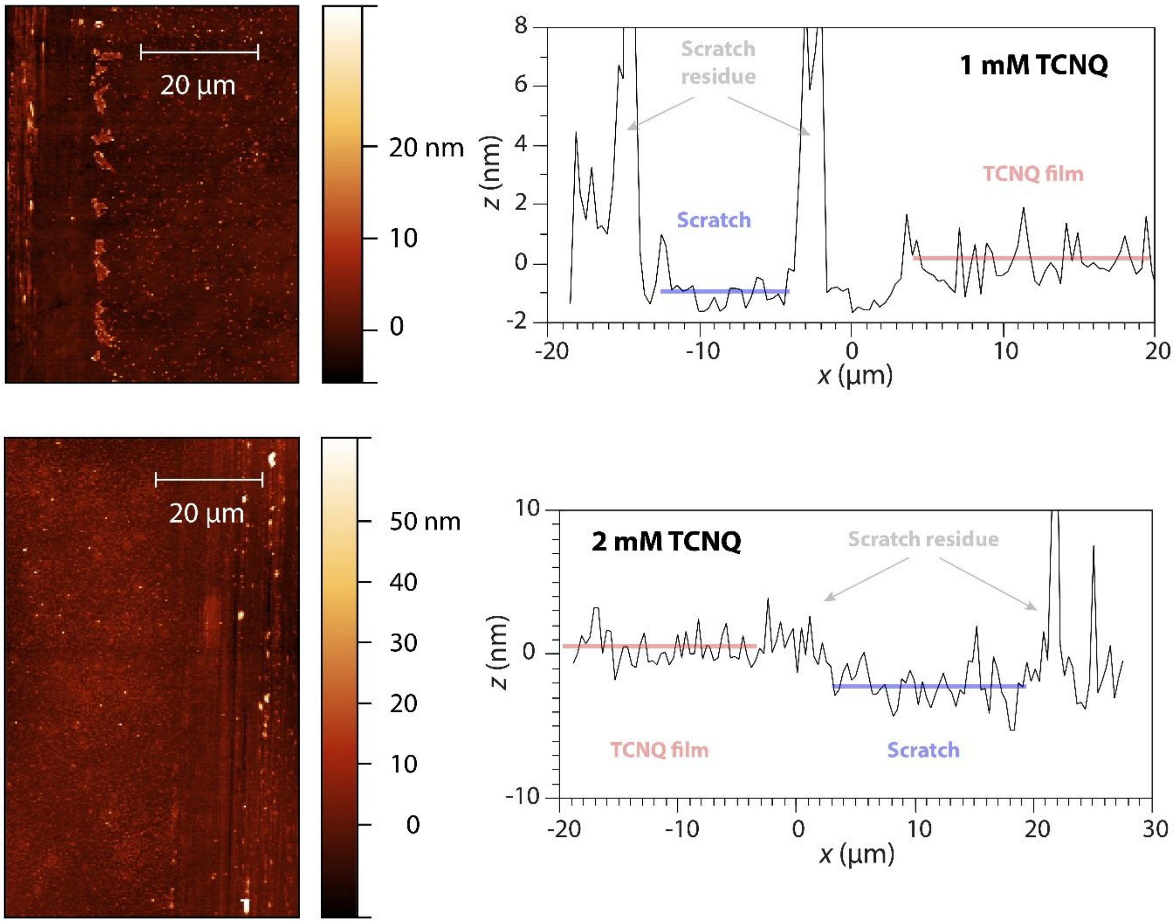

Supporting Figure S4 | AFM images of TCNQ on amorphous quartz used to estimate TCNQ thickness. AFM images and examples horizontal cross sections averaged over 5 consecutive lines. The top and bottom rows show data for two different TCNQ concentrations, where the sample was manually scratched after spin coating to create a step for height determination.

Supporting Section S4. Hyperspectral PL imaging

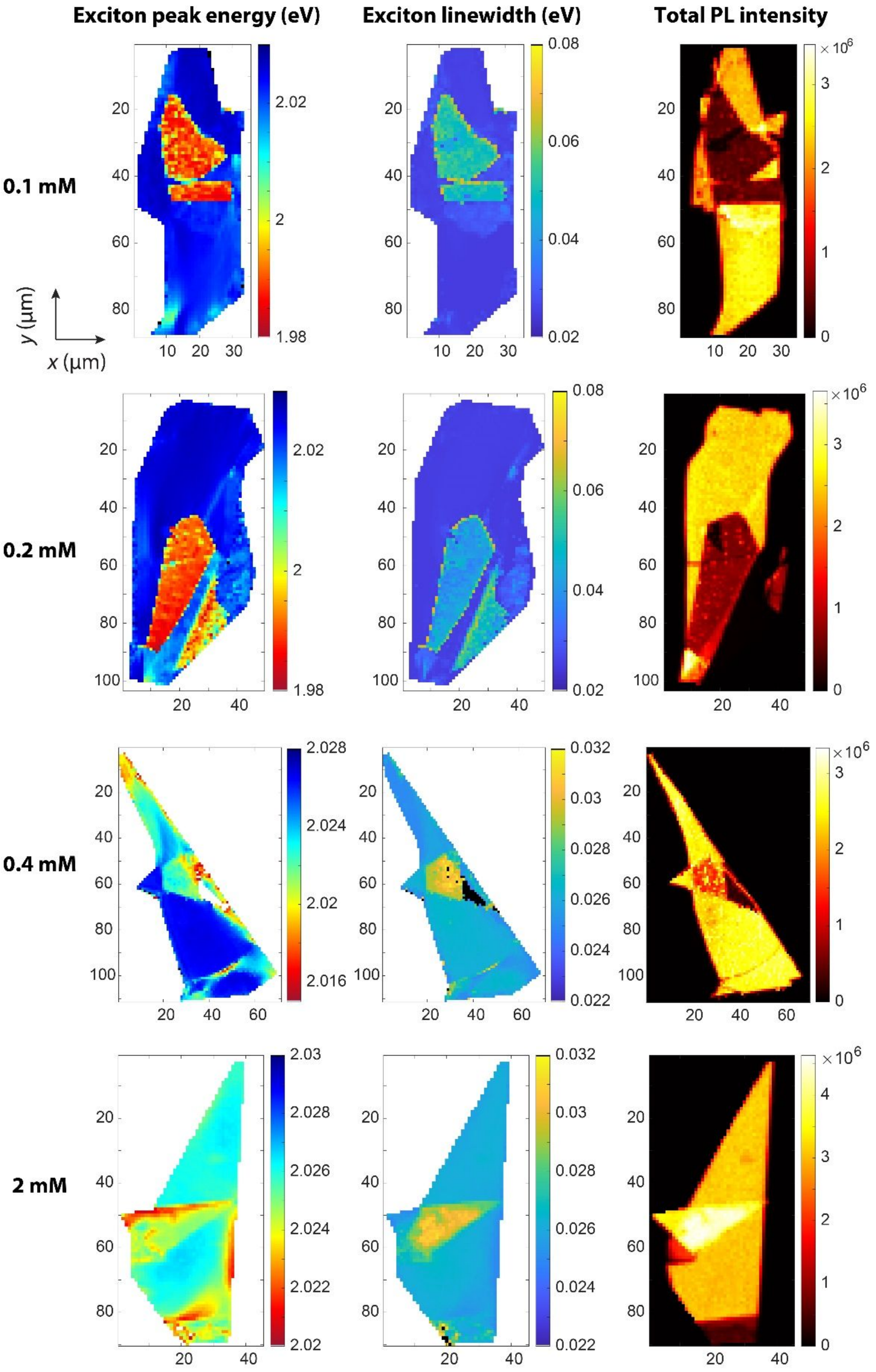

Supporting Figure S5 | Hyperspectral images of stacked WS_2 monolayers with TCNQ spacers. Hyperspectral image fitting results for the A-exciton peak energy and linewidth, and total spectrally

integrated PL intensity. The TCNQ concentration increases from the top to the bottom rows. The data corresponds to the scatter plot in Figure 3.
